# Unveiling End-of-Life Disparities in Chronic Rheumatic Heart Disease: A 22-Year Analysis of the CDC-WONDER Database

**DOI:** 10.7759/cureus.75162

**Published:** 2024-12-05

**Authors:** Anuva Khanal, Nidhi Laxminarayan Rao, Anubama Rajaravichandran, Alen Antony Pathil, Kaustav Majumder, Jairon Rodriguez

**Affiliations:** 1 Internal Medicine, Shaheed Ziaur Rahman Medical College and Hospital, Bogra, BGD; 2 Internal Medicine, K.A.P.Viswantham Government Medical College, Tiruchirappalli, IND; 3 Obstetrics and Gynaecology, PSG Institute of Medical Sciences and Research, Coimbatore, IND; 4 Obstetrics and Gynaecology, Coimbatore Medical College, Coimbatore, IND; 5 Internal Medicine, Sri Devaraj Urs Medical College, Kolar, IND; 6 Internal Medicine, R.G. Kar Medical College and Hospital, Kolkata, IND; 7 Cardiology, St. George's University School of Medicine, Port St. Lucie, USA

**Keywords:** cardiology, cdc wonder, chronic rheumatic heart disease, database, disparities, end-of-life care, mortality, place of death, research, retrospective

## Abstract

Background

This research examines mortality patterns and the place of death in individuals with chronic rheumatic heart disease (RHD) in the United States, aiming to identify demographic predictors for home or hospice death. Additionally, the study aims to uncover trends in mortality due to RHD and provide a predictive forecast.

Methods

The study utilized data from the Centers for Disease Control and Prevention (CDC)-Wide-Ranging Online Data for Epidemiologic Research (WONDER) database, which spans 22 years (1999-2020), and was categorized based on place of death, including home or hospice care, inpatient, outpatient, or emergency room deaths, and nursing home facility deaths. The data was further analyzed by age, gender, race, and region. The Autoregressive Integrated Moving Average (ARIMA) model was used for statistical analysis and forecasting.

Results

A total of 73,673 deaths were analyzed, and age was found to be a significant predictor of place of death. The highest number of deaths was in the 85+ age group, followed by a decrease in likelihood with decreasing age. Individuals residing in the West were more likely to die at home or in hospice compared to those in other regions. White individuals had a higher likelihood of dying at home or in hospice compared to other racial groups.

Conclusions

The findings emphasize the importance of considering patients' preferences and ensuring equitable access to end-of-life care services, regardless of their demographic background. The study highlights the need for further research to improve access to palliative care, reduce disparities in end-of-life care, and enhance the quality of life for individuals with chronic RHD and their families.

## Introduction

Around seven thousand people in the United States die from a variety of diseases every day, and healthcare providers will inevitably encounter patients who are actively dying at some point in their careers, making it crucial to understand the clinical significance and disparities in end-of-life care. The National Hospice and Palliative Care Organization (NHPCO) defines end-of-life care, or hospice care, as the point from when a person has a terminal illness with less than six months to live, and curative treatment options are no longer viable. Early conversations about end-of-life care should take place to allow sufficient time for incorporating patients' values, goals, and preferences into their care [1]. Although patients prefer to die at home, surrounded by familiarity and the presence of loved ones, various challenging factors such as insufficient nursing care, exhausted family caregivers, and financial constraints often lead to hospital care being prioritized [2].

Rheumatic heart disease (RHD) is the most common cause of cardiovascular morbidity and early mortality among young patients worldwide, often resulting from Group A streptococcal (GAS) infections [3]. A retrospective analysis of individuals aged 25 and older revealed that 141,137 deaths related to RHD occurred between 1999 and 2020. Of these deaths, 140,825 had a known place of death, with 56.4% occurring in hospitals, 33.1% in the decedent's home, and the remaining in nursing homes and long-term care facilities [3]. However, there is limited literature available on trends in place of death, which could help identify any disparities faced by patients in end-of-life care.

Aims and objectives

The main aim of this research is to evaluate and quantify the differences in the home or hospice death and medical facility or nursing home death for individuals with chronic rheumatic heart disease in the USA between 1999 and 2020, taking into account four parameters: age group, gender, race, and the four census regions of the country. Additionally, the study aims to identify trends and patterns in the distribution of death among different locations in order to shed light on potential disparities and the factors that influence end-of-life care decisions for those with chronic rheumatic heart disease.

## Materials and methods

The study relied on the Centers for Disease Control and Prevention (CDC)-Wide-Ranging Online Data for Epidemiologic Research (WONDER) database. This database compiles data from the National Center for Health Statistics and provides comprehensive mortality statistics derived from the death certificates of patients across the United States of America. As the CDC-WONDER database is a publicly available, free-to-use database, the present study was deemed exempt from ethical approval.

Data was obtained on February 26, 2024, and encompassed all fatalities resulting from chronic rheumatic heart disease between 1999 and 2020, utilizing the Bridged-Race categories. The International Classification of Diseases, Eleventh Revision (ICD-11), World Health Organization (WHO) 2019/2021 https://icd.who.int/browse11 code "105-09" was employed to identify all pertinent cases [4].

The study was conducted at the following institutes: R.G. Kar Medical College and Hospital (Kolkata), K.A.P. Viswanatham Government Medical College (Periyamilaguparai), and Coimbatore Government Medical College and PSG Institute of Medical Sciences and Research (Coimbatore). The subsequent analysis focused on the total number of fatalities within the specified timeframe, categorized by location of death, such as home or hospice care, medical facility death (including inpatient, outpatient, and emergency room deaths), death upon arrival, and status unknown, nursing home facility death, and others (including locations other than these or unknown).

The data was further subdivided based on the patient's 10-year age ranges, gender, race (Asian or Pacific Atlanta, American Indian or Alaska Native, White, Black, or African American individuals), and U.S. census areas (Northeast, Midwest, South, and West).

The collected data was exported to Microsoft Excel (Microsoft Corporation, Redmond, Washington, United States) for further analysis. Missing data, if present, was handled using listwise deletion, meaning cases with missing or incomplete information on key variables were excluded from the analysis. The decision to exclude missing data was based on the completeness of the CDC-WONDER dataset, where missing entries were relatively rare. This approach was chosen to ensure consistency in the analysis and avoid biases that imputation methods might introduce.

Statistical analysis and data visualization were performed using an Autoregressive Integrated Moving Average (ARIMA) model. For the analysis of temporal trends, an Autoregressive Integrated Moving Average (ARIMA) model was used. The ARIMA model is a widely-used tool for time-series forecasting, particularly in epidemiology, as it accounts for trends, seasonality, and autocorrelations in longitudinal data. The model was chosen for its ability to describe the trend in mortality over the 22-year period and provide forecasts for potential future trends.

Statistical tests included univariate logistic regression in order to assess the differences in the place of death according to patient demographics. A p-value <0.05 was considered statistically significant.

## Results

Aggregate data of 73,673 deaths from 1999-2020 was obtained for chronic rheumatic heart disease (RHD) from the CDC-WONDER database.

Table 1 depicts the total number of deaths based on place for chronic rheumatic heart disease from 1999 to 2020. The age group with the lowest number of deaths in home or hospice care was between <1 to 14 years (0%; n=0), while the age group with the highest number of deaths was 85 years and older (42.5%; n=7,892). The age group of one to four years recorded the fewest deaths in medical facilities or nursing homes (0.05%; n=24), whereas the age group of 75-84 years had the most deaths (30.7%; n=16,141). For the 'Others' location, the age group with the least number of deaths was between <1 to 24 years (0%; n=0), and the age group with the most deaths was above 85 years (48.1%; n=1,197).

**Table 1:** Total number of deaths in each place of death, according to demographic variables Values have been reported as N values.

Variables (n)	Home or Hospice	Medical Facility or Nursing	Others
Ten-Year Age Groups	(n =18590)	(n =52595)	(n =2488)
< 1 year (n = 46)	0	46	0
1-4 years (n = 24)	0	24	0
5-14 years (n = 58)	0	58	0
15-24 years (n = 222)	16	206	0
25-34 years (n = 878)	107	742	29
35-44 years (n = 1,768)	236	1493	39
45-54 years (n = 4,043)	679	3271	93
55-64 years (n = 7,930)	1353	6390	187
65-74 years (n = 13,625)	2634	10716	266
75-84 years (n=22,491)	5673	16141	677
85+ years (22,597)	7892	13508	1197
Gender	(n =18593)	(n =52643)	(n =2507)
Female (n = 49,913)	12604	35508	1801
Male (n= 23,830)	5989	17135	706
Census Region	(n =18593)	(n =52638)	(n =2499)
Census Region 1: Northeast (n = 14,073)	3517	10287	269
Census Region 2: Midwest (n = 18,273)	4196	13433	644
Census Region 3: South (n = 22,597)	5255	16491	851
Census Region 4: West (n = 18,787)	5625	12427	735
Race	(n =18588)	(n =52639)	(n =2501)
American Indian or Alaska Native (n = 433)	80	335	18
Asian or Pacific Islander (n = 2215)	467	1703	45
Black or African American (n = 5,870)	1025	4703	142
White (n = 65,210)	17016	45898	2296

In terms of gender, there were more deaths overall in females (67.7%; n=49,913) than in males (32.3%; n=23,830). Medical facilities or nursing homes were the locations with the highest number of deaths for both females (71.1%; n=35,508) and males (71.9%; n=17,135).The least number of deaths occurred in the 'Others' locations for both females (3.6%; n=1,801) and males (3%; n=706).

In terms of census region, the South region had the highest overall number of deaths (30.7%; n=22,597), of which the maximum number of deaths occurred in medical or nursing facilities (73%; n=16,491). The Northeast region had the lowest overall number of deaths (19.1%; n=14,073), with the highest number of deaths occurring in medical or nursing facilities (72.1%; n=10,287).

Based on race, the overall maximum number of deaths was attributed to White patients (88.5%; n=65,210), while the overall minimum was attributed to American-Indian or Alaska Native patients (3%; n=2,215). In all races, the maximum number of deaths occurred in medical or nursing facilities.

Table 2 shows the predictors of home or hospice deaths for chronic rheumatic heart disease from 1999 to 2020. It was observed that individuals aged 75-84 years, male gender, patients residing in the West region, and White patients were more likely to die in home or hospice care, as predicted via univariate logistic regression (p <0.05).

**Table 2:** Univariate logistic regression of place of deaths according to demographic variables Values are reported as odds ratios and confidence intervals (N); p values <0.05 was considered to be statistically significant.

Variables	Univariate Logistic Regression		
	Odds Ratio	95% Confidence Interval	P-value
Age			
< 1 year	0	(0, 3.751e+61)	0.87
1-4 years	0	(0, 2.551e+87)	0.906
5-14 years	0	(0, 1.661e+54)	0.854
15-24 years	0.145	(0.087, 0.241)	<0.001*
25-34 years	0.259	(0.211, 0.317)	<0.001*
35-44 years	0.287	(0.25, 0.33)	<0.001*
45-54 years	0.376	(0.345, 0.41)	<0.001*
55-64 years	0.383	(0.359, 0.409)	<0.001*
65-74 years	0.447	(0.425, 0.47)	<0.001*
75-84 years	0.629	(0.603, 0.655)	<0.001*
85+ years	1.0 (Reference)		
Gender			
Male	0.994	(0.959, 1.03)	0.726
Female	1.0 (Reference)		
Census Region			
Census Region 1: Northeast	1.000 (Reference)		
Census Region 2: Midwest	0.895	(0.85, 0.942)	<0.001*
Census Region 3: South	0.909	(0.866, 0.955)	<0.001*
Census Region 4: West	1.283	(1.221, 1.348)	<0.001*
Race			
Asian or Pacific Islander	1.263	(1.117, 1.427)	<0.001*
American Indian or Alaska Native	1.071	(0.833, 1.378)	0.592
White	1.669	(1.557, 1.789)	<0.001*
Black or African American	1.000 (Reference)		

Moreover, it was also observed that those aged 85 years and above have the highest probability of dying in home or hospice care, while those aged between <1 year to 14 years have the least probability. Males are 0.994 times less likely to die in home or hospice care than females, and patients from the West are 1.263 times more likely to die in home or hospice care than those from the Northeast. Patients from the Midwest, on the other hand, are 0.895 times less likely to die in home or hospice care. White patients have a 1.669 times higher likelihood of death in home or hospice compared to Black or African American patients.

**Figure 1:**
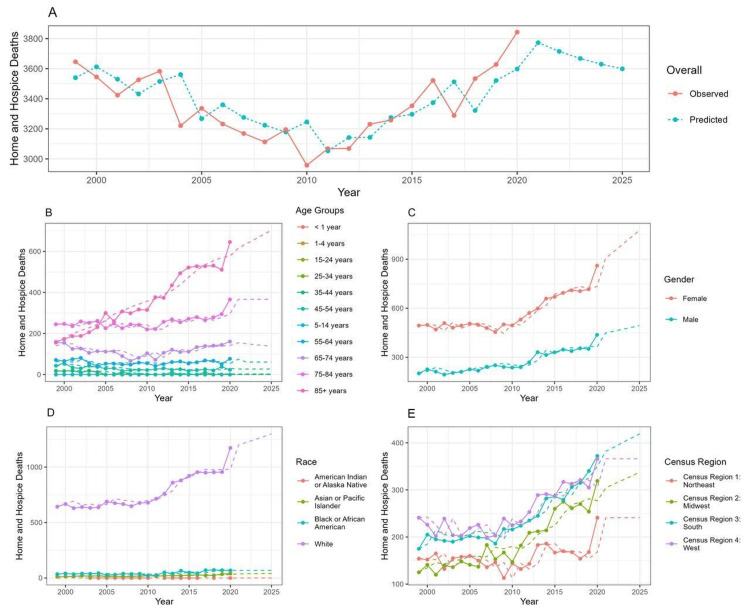
Predictive forecasting of death trends until 2025 A: overall trends in mortality (observed and predicted); B: mortality trends according to 10 year age groups; C: mortality trends according to gender; D: mortality trends according to race; E: mortality trends according to census region

Figure 1A shows that the overall deaths from chronic rheumatic heart disease in home or hospice care are increasing, with significant decreases in 2001, 2004, 2010, and 2017. Figure 1B indicates that individuals aged 85 and above have an increasing trend in deaths, while those aged between 25 and 44 years have remained constant over the years. Figure 1C reveals that female deaths are higher than male deaths. Based on figure 1D, it can be determined that White patients have the highest death rates, with the trend gradually increasing in comparison to other races. Finally, Figure 1E indicates that the West has the highest death rate, with the trend gradually increasing compared to other regions.

## Discussion

Twenty-two years of data (1999-2020) were collected from CDC-WONDER to study mortality patterns related to rheumatic heart disease, encompassing a total of 73,673 deaths. This data included 18,593 (25%) home or hospice deaths, 52,643 (71.5%) medical facility deaths, and 2,507 (3.4%) deaths categorized as ‘Others.’ Leveraging this data, we generated reliable predictions for an additional five years, bolstering the strength and reliability of our findings. This extended time frame allows for a more comprehensive understanding of the disease's long-term trends and potential future developments.

This study investigated the mortality trends and place of death in patients suffering from chronic rheumatic heart disease. A greater number of deaths occurred in medical facilities and nursing homes compared to home or hospice settings. This aligns with findings by Siddiqui et al., who observed disparities in place of death among diabetic patients [5]. Moreover, our analysis identified age, region, and race as significant predictors of home or hospice death. This emphasizes the critical need for ensuring comprehensive and accessible end-of-life care options for RHD patients, irrespective of their demographic background.

Consistent with existing knowledge, the study also found that mortality rates for individuals with RHD climb steadily with age, with the highest number of deaths observed in the 85+ age group [6]. The highest number of home and hospice deaths was observed to occur among the 85-year-old and above age group. These environments offer several benefits, including reduced need for medical interventions, fostering a warm and intimate atmosphere for quality time with loved ones, and empowering patients with greater control over their surroundings [7].

The probability of passing away at home or in hospice care decreases as age decreases, in contrast to individuals who are 85 years old or older. However, this likelihood increases with age until the 75-84 age group. This result aligns with a study conducted by Cross et al., who also similarly found a lower likelihood of hospital death and a higher likelihood of dying in a nursing home or hospice with advancing age, compared to those under 65 [8]. These findings differ from the study by Al Hussein et al., who found a higher likelihood of home or hospice death in the 45-54-year-old age group [9]. This is consistent with the results of Siddiqui et al., who found the highest probability of home or hospice death in the age group of 55 to 64 years [10].

This study also revealed that there were more female deaths than male deaths in all categories. This finding is consistent with existing data that show a higher global burden of RHD among females, reflected in both higher prevalence and mortality rates [6]. Although our results suggest that gender does not significantly influence the likelihood of dying at home or in hospice care, several other studies have shown an increased probability of male deaths in home or hospice care [5,10].

Our study uncovered significant regional discrepancies in end-of-life care for RHD patients. The South had the highest number of fatalities, while individuals in the West were more likely to pass away at home or in hospice facilities compared to those living in the Northeast, Midwest, and South. This finding aligns with some previous research (Siddiqui et al.), but contrasts with others (Hussein et al.), who reported a higher likelihood of home/hospice deaths in the South [5,9]. The observed regional disparities in end-of-life care for RHD might be influenced by various factors, such as differences in population characteristics, healthcare resource availability, and existing economic disparities across different regions of the country [5]. Notably, countries with well-developed healthcare systems often have lower hospital death rates, likely due to the availability of alternative care options outside of hospitals, allowing individuals to spend their final moments in a more familiar and comfortable environment. This underscores the importance of ensuring accessible and comprehensive end-of-life care options for RHD patients across all regions [11].

Our study further revealed significant racial disparities in the place of death for RHD patients. Although the overall mortality rate was highest among White individuals, those who identified as Black or African American individuals had a considerably lower likelihood of passing away at home or in hospice care compared to those who identified as White, Asian, or Pacific Islander, American Indian, or Alaska Native individuals. Interestingly, White individuals showed the highest odds of dying at home or in hospice. These findings highlight the concerning presence of racial disparities in end-of-life care for RHD patients. Furthermore, studies by Chuzi et al. and Cross et al., investigating trends in cardiovascular mortality, report similar patterns of racial disparities in place of death [8,12]. Several studies have identified a potential connection between race and ethnicity and the utilization of hospice care. Research suggests that areas with a larger percentage of Black and Hispanic residents tend to have lower hospice use rates compared to other demographics [12]. Additionally, another report found a similar trend, where White individuals were more likely to experience cardiovascular death in a hospice facility or at home compared to their Black counterparts [13]. These observed racial disparities might be attributed to a combination of complex factors, including potential biases in healthcare access and treatment, as well as unfavorable social and economic determinants impacting health outcomes [14]. Furthermore, research conducted by Wang et al. has consistently identified statistically significant differences in hospice utilization between African American and White patients [15]. These disparities in hospice use extend beyond just Black or African American individuals. Research by Ngo-Metzger suggests that both U.S.-born and foreign-born Asian residents were less likely to use hospice than the White population, highlighting the need for further exploration of factors influencing disparities within and between racial and ethnic groups [16].

Meeting patients' preferred place of death (PPOD) is consistently identified as a crucial indicator of high-quality palliative care. Research emphasizes the importance of respecting patients' choices, considering it an essential component of optimal terminal care [16,17]. Honoring these preferences demonstrably improves the quality of end-of-life services and fosters greater social equality [18]. This viewpoint is consistent with that of healthcare policymakers, who acknowledge the significance of comprehending PPOD. Emphasizing PPOD promotes improved end-of-life care, demonstrates adherence to patient preferences, and streamlines resource allocation to ensure the delivery of effective palliative care [19,20].

Various other studies also consistently highlighted home as the chosen place of death for the majority of people, including terminally ill patients [21-26]. A systematic review by Bell et al. (2010) confirms this global trend, revealing that the home is the most favored location for cancer patients worldwide [27]. This preference stems from the inherent comfort and familiarity of one's own home, allowing individuals to spend their final moments surrounded by loved ones [28]. A systematic review by Hoare et al. further supports this notion, demonstrating that when missing data points were excluded, the majority of participants expressed a desire to die at home [22]. Interestingly, other findings by Lee et al. suggest no significant difference in place of death preference based on a patient's specific diagnosis [29]. However, it's crucial to acknowledge the gap between preferences and realities.

Despite a strong preference for home-based end-of-life care, a separate study by Iwashyna et al. (2015) found that over two-thirds of patients ultimately passed away in hospitals, with this proportion continuing to rise [30]. Various factors influence the alignment between the desired and actual place of death. Studies examining this "congruence" identify supportive factors like physician support, hospice enrollment, and family assistance, while hospitalization, lack of family support, and inadequate symptom control can hinder it [27].

Disparities in place of death exist based on sociodemographic factors. Research shows that Black and Hispanic individuals are more likely to die in hospitals compared to White individuals, who lean towards nursing homes, even after accounting for other variables like age and income [30,31]. Additionally, married patients have a higher chance of dying at home due to a larger potential caregiver pool, while those with higher education and income benefit from greater access to resources, also increasing their likelihood of home-based death [8,30].

It was also observed that the available literature demonstrates a positive association between receiving palliative care and dying at home, indicating that palliative care plays an important role in influencing the place of death. Patients receiving palliative care were twice as likely to die at home as those who did not receive such care, according to one study by Quinn et al. [32]. Moreover, the provision of palliative care services in diverse environments, including residential care facilities and nursing homes, augments the probability of passing away in said specific locations as opposed to a medical facility [32]. Conversely, hospital-based end-of-life care increases the chance of dying in a hospital compared to home [33].

To provide the best end-of-life care and support services for individuals with chronic RHD, it is crucial to comprehend the factors influencing their place of death. Healthcare practitioners should consider the distinctive needs and preferences of patients and their families when discussing treatment alternatives and planning for end-of-life care. Future research should concentrate on creating treatments that boost access to palliative care services, reduce inequities in end-of-life care outcomes, and improve the quality of life for individuals with chronic RHD and their families.

Limitations

Our analysis covers data from 1999 to 2020, excluding the recent period of 2021-2023 due to its unavailability in the CDC-WONDER database. This restricts our ability to capture the most recent trends and potential shifts in mortality patterns or places of death. Future research could revisit the study when the latest data becomes accessible. While we analyze broad categories like home/hospice, medical facility, and others, the study doesn't delve into specific subcategories within these categories. Examining locations like nursing homes or specific hospital types could provide a more nuanced understanding of end-of-life care utilization, especially within the "others" group. Our study further does not account for socioeconomic factors such as income, education, or access to healthcare, which are significant contributors to disparities in end-of-life care. These factors often correlate with both healthcare access and quality and may influence the place of death for different demographic groups. The study also relies solely on data from CDC-WONDER, which utilizes information from death certificates. While valuable, death certificates may contain inaccuracies or inconsistencies in completion, potentially affecting the accuracy and reliability of the data. Future research could explore alternative data sources, like collaborating with healthcare providers, to potentially improve data robustness.

## Conclusions

The study findings underscore several noteworthy trends in the distribution of deaths among individuals with chronic rheumatic heart disease. Firstly, the majority of deaths occur within medical facilities, highlighting a reliance on institutionalized care for end-of-life management. Notably, older individuals consistently contribute the highest number of deaths across both medical facilities and hospices, suggesting the need for further investigation into factors such as sedentary lifestyle, nutritional status, and geriatric health concerns that may contribute to mortality in this demographic. Additionally, gender disparities in mortality rates are evident, with females exhibiting a higher number of deaths compared to males, potentially influenced by hormonal, anatomical, and lifestyle factors. Furthermore, regional disparities in death rates suggest that climate, economic conditions, and lifestyle differences among census regions may contribute to variations in mortality rates, emphasizing the need for nuanced analyses to understand the underlying factors driving these trends. Moreover, racial disparities in mortality rates highlight the role of genetic factors in shaping disparities in death rates among different racial groups, warranting further exploration to elucidate the complex interplay between genetics, healthcare access, and health outcomes.

The findings from this research provide important insights that can guide future healthcare interventions and research. By understanding the factors that contribute to mortality among older individuals, particularly those with chronic rheumatic heart disease, targeted interventions can be developed to improve geriatric health outcomes and enhance end-of-life care for this demographic. Furthermore, investigating the mechanisms underlying gender disparities in mortality rates can inform tailored approaches to address gender-specific health concerns and mitigate disparities in healthcare access and outcomes. Addressing regional disparities in death rates requires targeted interventions that address socio-economic factors, improve healthcare infrastructure, and promote healthy lifestyles across different geographic regions. Moreover, efforts to address racial disparities in mortality rates necessitate a multifaceted approach that addresses systemic barriers to healthcare access, promotes culturally sensitive care, and addresses underlying socio-economic determinants of health. In conclusion, these findings highlight the importance of addressing disparities in end-of-life care and health outcomes to ensure equitable access to quality healthcare for all individuals with chronic rheumatic heart disease, regardless of their age, gender, race, or geographic location.
